# The programming of sequences of saccades

**DOI:** 10.1007/s00221-019-05481-7

**Published:** 2019-02-06

**Authors:** Eugene McSorley, Iain D. Gilchrist, Rachel McCloy

**Affiliations:** 1 School of Psychology and Clinical Language Sciences, University of Reading, Reading, RG6 6AL UK; 2 School of Experimental Psychology, University of Bristol, Bristol, BS8 1TU UK

**Keywords:** Saccade, Sequences, Parallel programming, Eye movements

## Abstract

Saccadic eye movements move the high-resolution fovea to point at regions of interest. Saccades can only be generated serially (i.e., one at a time). However, what remains unclear is the extent to which saccades are programmed in parallel (i.e., a series of such moments can be planned together) and how far ahead such planning occurs. In the current experiment, we investigate this issue with a saccade contingent preview paradigm. Participants were asked to execute saccadic eye movements in response to seven small circles presented on a screen. The extent to which participants were given prior information about target locations was varied on a trial-by-trial basis: participants were aware of the location of the next target only, the next three, five, or all seven targets. The addition of new targets to the display was made during the saccade to the next target in the sequence. The overall time taken to complete the sequence was decreased as more targets were available up to all seven targets. This was a result of a reduction in the number of saccades being executed and a reduction in their saccade latencies. Surprisingly, these results suggest that, when faced with a demand to saccade to a large number of target locations, saccade preparation about all target locations is carried out in parallel.

## Introduction

To carrying out everyday visual tasks in the most efficient and effective way possible, we make multiple sequential eye movements (Hayhoe and Ballard 2005; Land 2006). The most common eye movements we make are those that move the eyes quickly from one location to another. The majority of research dedicated to understanding the control of saccadic eye movements is concerned with isolated single saccadic eye movements in response to target and distractor configurations while relatively little work has been carried out examining their control when they form part of the sequences of multiple movements.

The parallel programming of multiple saccades has long been suggested with both behavioral and physiological studies providing evidence that the saccadic system may be able to program at least two or three responses in parallel (Becker and Jurgens 1979; Godijn and Theeuwes 2002; McPeek et al. 2000; Theeuwes et al. 1998). However, many models of saccade generation have not been developed with this in mind and are largely designed to account for individual responses. They generally adopt a “winner-take-all” approach with competition between different potential saccade targets taking place before culminating in a single saccade response (Findlay and Walker 1999; Trappenberg et al. 2001).

The saccadic eye movements made in common everyday behaviors such as sandwich making (Hayhoe et al. 1998), tea making (Land et al. 1999) or driving (Land 1998) show scan paths that contain many movements to objects and locations that only come into play at later points in the task suggesting that information may be processed in parallel to sequence movements. During reading, saccadic eye movements are also used to move the eye across the text in short jumps. Here the evidence suggests that word information (such as its visual features) is programmed in the parallel (see Rayner 2009 for a review). Indeed some of the more successful models of reading incorporate elements of parallel processing in their architecture (Engbert et al. 2005; Reichle et al. 2012).

Evidence for parallel programming of saccades has also been found in simple laboratory-based tasks. Here the control and execution of multiple saccadic eye movements has been examined using saccade sequences, double- or triple-step tasks or via corrective secondary saccades following initial error responses (Hooge and Erkelens 1996; Mokler and Fischer 1999). Participants are asked to saccade sequentially in response to a set of visual targets that are on screen at all times box to box, to a set of green circles in amongst red ones, or directed by oriented Landolt C’s (Gersch et al. 2004; Gersch et al. 2009; Turkenbrod and; Engbert 2012) or to targets that have been memorized such that subsequent saccades are memory-guided (Gersch et al. 2009). In double-step and triple-step tasks, participants are asked to make two or three saccades in response to a predetermined set of instructions, e.g., “saccade to a target and then once more to the target two positions clockwise of it” (Baldauf and Deubel 2008); or “execute two saccades when fixation is removed in response to a target which has stepped from location to another” (Bhutani et al. 2012, 2013).

Across all of these studies, the evidence consistently supports the position that information about saccade target locations beyond simply the next one is processed in parallel. This evidence takes the form of a performance improvement or of an impact on the underlying processes involved in the control of saccadic responses. For example, performance improvements have been found in identification tasks when targets are shown at future saccade target locations relative to other non-target locations (Baldauf and Deubel 2008; Gersch et al. 2009). These results have been interpreted within an attentional framework with enhancement at future saccade target locations caused by the parallel spread of attention along the sequence of saccade target locations with consequent benefits on saccade programming. This benefit is modulated by task demands and diminishes the further the future saccade target is from current fixation position (Baldauf and Deubel 2008; Gersch et al. 2004; Gersch et al. 2009). Within this context, it has also been suggested that the parallel programming of saccade sequences may take place in different reference frameworks, either retinocentric or oculocentric depending on whether they are executed between or within objects and whether they are executed quickly (Lavergne et al. 2008; Vergilino and Beauvillain 2001; Vergilino-Perez and Findlay 2003, 2006).

Further evidence for saccades being programmed in parallel has been shown in reports of very short latencies for secondary corrective saccades following initial error responses. In the anti-saccade task, saccades made in the direction opposite to a peripheral stimulus onset (Hallet 1978) sometimes produce erroneous reflexive saccades made to the peripheral stimulus (around 10–15% ‘pro-saccade errors’) and that are followed by secondary corrective saccades after a very brief inter-saccadic fixation period (0–100 ms) (Amador et al. 1998; Mokler and Fischer 1999; Weber et al. 1998). Corrective saccades following very short fixation intervals following error responses have also been found in visual search tasks after distractor-directed saccades are corrected (Findlay et al. 2001; Godijn and Theeuwes 2002; Hooge and Erkelens 1996; McPeek et al. 2000; Theeuwes et al. 1998; Vivani and; Swensson 1982). It is argued that such very short inter-saccadic fixation periods are only possible if the second saccade has already been prepared.

As well as shortening saccade latency responses, reports have also been made of an effect of second target location on metrics of first saccade response suggesting that some aspects of the metrics of saccades can be programmed in parallel. The landing positions of first saccades have been shown to deviate between two target positions when a saccade to each is required suggesting an “averaging” or at least an interference between the programs. The second target location has also been shown to influence the trajectories of first saccade movements (Godijn and Theeuwes 2002; Bhutani et al. 2012, 2013). Parallel programming of saccades is also suggested from a consideration of the effect of making sequences of saccades on the compression of visual space (Zimmerman et al. 2014; Zimmerman 2015).

A series of studies has examined instances where the task explicitly demands two or three saccades are executed. Here the common finding is of latency reduction of the second and third saccades (i.e., brief fixations or inter-saccadic intervals), when multiple saccades are required (De Vries et al. 2014; McSorley et al. 2016; Walker and McSorley 2006). Walker and McSorley (2006) showed latency reductions of the second saccade response made as part of a chain of either voluntary then reflexive saccade sequences or reflexive then voluntary saccade sequences. Following this, McSorley et al. (2016) varied the position of the second target locations during the flight of the first response to examine the extent that the metrics of multiple saccades are programmed in parallel. They found that the latency of the responses was shortened and the distance and direction of the second movement deviated towards the original position of the second target. These results were interpreted in the context of a general framework in which there are three distinct but interconnected stages involved in the processing of goal directed saccades: a visual saliency stage in which bottom-up sensory encoding of stimuli takes place, the goal of which is to compute a saliency map (Itti and Koch 2000); an intermediate stage that combines saliency information with top-down goal demands and selection history to produce a common priority map of movement goals (Awh et al. 2012; Fecteau and Munoz 2006); and finally a motor stage in which motor representations are generated to produce eye movements. In this framework, sequential saccadic eye movements are continuously programmed in parallel with new saliency and goal information processed and combined to produce each individual eye movement. In this context, the parallel influence of saccade programs on one another is dependent on the rate at which their programs develop. More quickly developing future saccade programs will have greater influence on preceding movements.

In summary, evidence from a variety of sources, whether laboratory-based or during everyday tasks, supports the position that saccadic eye movements are not programmed in a serial individual fashion but rather are the result of on-going parallel programming of the location of upcoming saccades which has an impact on the dynamics and metrics of the individual movements and leads to improvements in performance. The extent to which parallel programming of saccades takes place along a set of multiple targets appears to be limited with performance benefits found up to the next three targets (Baldauf and Duebel 2008). However, the demand to make saccades beyond the next few saccades has not previously been examined and consequently, it is unclear if the parallel programming of saccades can stretch beyond this to multiple target locations. Here we introduce a task in which multiple saccades are made in response to seven targets (small spots on a computer display). On each trial, targets are displayed simultaneously or their location is controlled and revealed during participants’ response such that the location of the next one, three or five target(s) is available. In this way, the participants’ prior information about the location of subsequent saccade targets is restricted. If parallel programming occurs across multiple saccade targets, then restricting this prior information should lead to an impact on saccade control. For example, if parallel programming is limited to the next three saccades, then having target location information beyond this (i.e., about the next five or all seven locations) should show no extra impact on saccade control.

## Method

### Observers

18 naïve observers participated in the experiment (12 females), aged between 18 and 25 years. All had normal, or corrected to normal eyesight. The University of Reading Ethics Board approved the ethics of this study, and the study was conducted in accordance with the standards described in the 1964 Declaration of Helsinki. Participants provided written informed consent. The authors declare that there is no conflict of interest.

### Apparatus

Participants’ eye movements (left eye only) were recorded using an Eyelink II, which is a head-mounted eye tracker with a 500 Hz sampling rate and a spatial resolution (RMS) of 0.025°. Participants placed their chin on a rest, which constrained any head movements and ensured the viewing distance remained at 1 m. Before the experiment began, the eye tracker was calibrated using a nine-point grid, and then validated using a different grid. Participants were allowed to begin the experiment when there was an average difference of less than 0.5 degrees between the actual eye position and that predicted from the calibration and the validation. Stimuli were presented on a 21” color monitor that had a refresh rate of 75 Hz.

### Stimuli

The fixation stimulus was a white “+” 1° in extent. The target stimuli were white circles (also 0.5°) overlaid with central black numbers labeling the targets (“1”–“7”; 0.35°). Each target was shown on the principal or oblique angles relative to preceding target at a 4° center to center separation. Stimuli were shown on a mid-gray background.

### Design

**Fig. 1:**
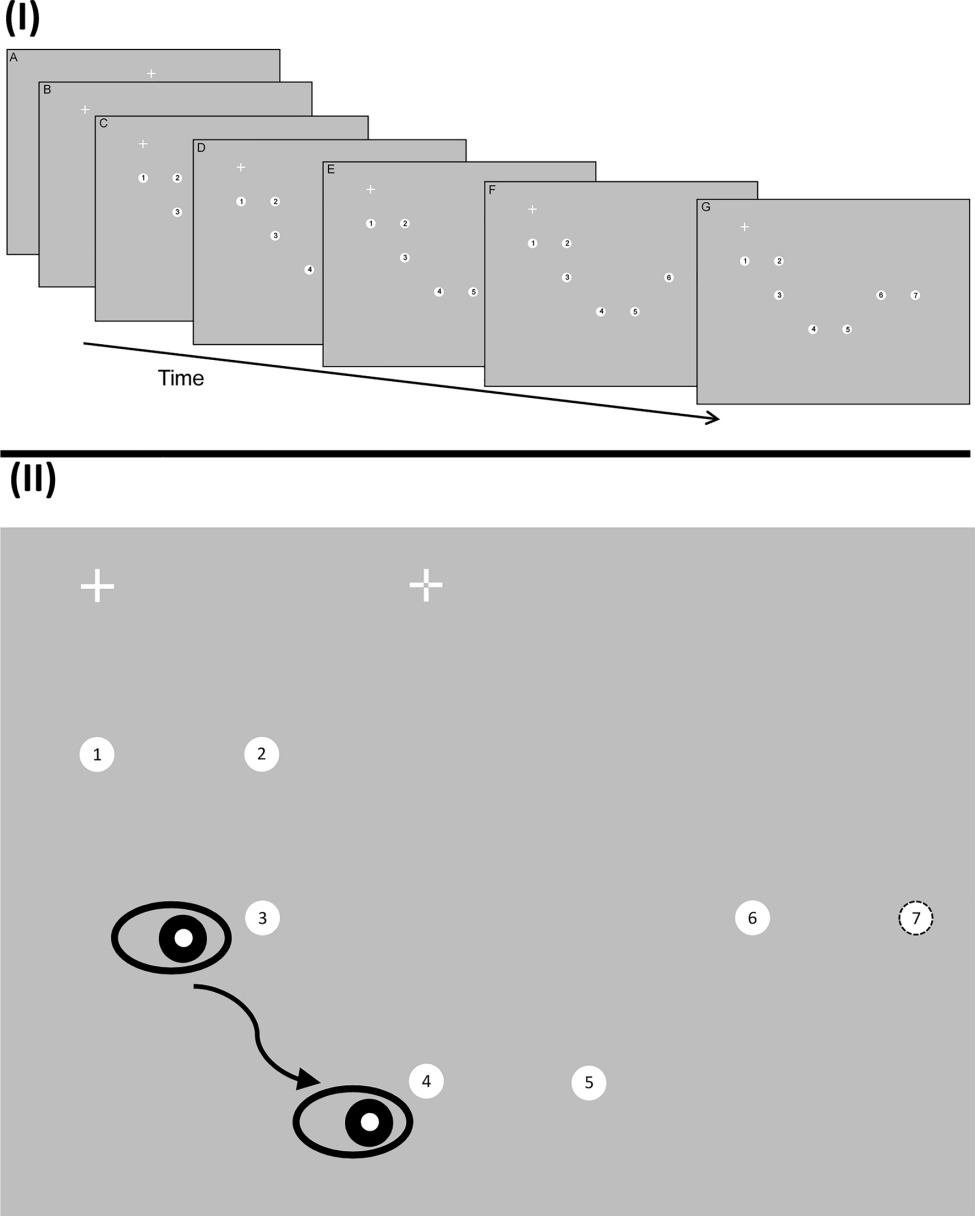
**I** Upper portion shows the run of displays revealed to the participant as they saccaded to each of the seven targets in turn (displays are shown overlapping to varying degrees for ease of visibility on the page). In this example, participants were shown the location of the next three targets relative to where they were currently fixated. (A) They initially fixated at random locations around the peripheral locations of the display. (B) Once fixated, the fixation cross was removed and reappeared 4° of visual angle away either on the vertical or horizontal axis. A number of targets (here 3) were also shown. (C–G) As participants saccade to each numbered target in turn the next target in the sequence was onset. **II** In the lower portion of the figure, the temporal unfolding of the same example sequence is illustrated. Participants initially fixated a central cross (shown here as dashed lines but actually solid when displayed). This fixation point disappeared then immediately reappear 10° to the left or right on the horizontal meridian (shown as a leftward movement here) to which a saccade was made. During this saccade the sequence is shown. The number of targets shown during the trial depended on the prior visual information level in that trial. As in the upper portion, the prior information level in this example is 3. In this trial, the participant was currently fixated on position 3 having already visited positions 1 and 2. Information about the next three target positions is given (positions 4, 5 and 6). The eye is shown as being in flight between positions 3 and 4. During this saccade, the target at position 7 is presented thus maintaining information about the next three target positions. The dashed outline circle is not shown in the actual display but illustrate the position of the remaining saccade target

Participants completed 84 trials in which the instruction was to saccade to seven targets in turn progressively numbered from 1 to 7 (see Fig. 1). The target locations and numbering were, to some extent, randomly generated but organized so the sequence naturally moved in a linear fashion, i.e., locations and numbering of the targets were shown so that they appear to progressively move away from the first target. Targets were positioned on the intersections of an unseen square lattice of potential target locations. This means that the targets always appeared on the main principal or oblique axes, with the vertical and horizontal intersections being 4.67° of visual angle in distance and thus the oblique locations being 6.47°. It is important to note that the target sequence never turned back on itself. This gave participants a sense that the sequence was naturally unfolding as they made their responses. Viewing distance was 1 m. The target display conditions were all seven targets were shown simultaneously; the next five were shown; the next three; or one target was displayed. The addition of new targets was made during saccade flight to take advantage of the reduction of visual sensitivity found during saccade suppression and thus minimize the disruption of new visual events on visual processing (Burr et al. 1982; Ross et al. 2001; Zimmermann et al. 2018). There were 21 trials per condition. Trial types were shown in a random order.

## Procedure

Participants were first familiarized with the stimuli and the task and were encouraged to carry out as many practice trials as they felt were necessary to become comfortable with the task and what they had to do. The calibration procedure was then carried out. Each trial started with a drift correction procedure in which a small spot was displayed offset from center by 6° of visual angle horizontally and 3° vertically and once fixated, eye position was accepted and automatic adjustments to the calibration were carried out by the eye-tracking software if the actual and expected eye position differed. Once accepted, a fixation cross was shown centrally for 800–1200 ms after which it “stepped” (was removed from display and then reshown) 10° of visual angle horizontally to the left or right and participants saccaded to the new position. During this saccadic response, targets were onset. Participants were asked to saccade to each target in turn. New targets were onset to the display during saccade responses as required until all seven were shown. The time at which new targets were onset was determined by a position criterion rather than a velocity one as it was found to be more stable, thus once the eye position crossed an invisible boundary set at 2° of visual angle from the center of the next target position (either the stepped location fixation cross or the next target) then the next new target was displayed. If the next target was not localized with sufficient accuracy then the trial effectively halts. On no occasion within the experiment did this happen; participants were successful in following instructions and their saccades were generally accurate as defined by this criterion. After each sequence of seven targets was fixated the trial ended and a new drift correction procedure was initiated before commencing the next trial.

### Data analysis

The eye tracking software includes a parser that was used to identify the start and ends of saccades using a 22 degree per second velocity and 8000° per second squared criteria (SR Research Ltd). Further analysis of trial durations, saccade counts and average latencies were accomplished offline using DataViewer (SR Research Ltd) to isolate individual saccades and in-house software analysis to calculate averages. To get a complete overview of control in the execution of saccade sequences, no exclusion criteria for saccades were adopted. All saccades were accepted as being a legitimate response to the target sequences. A number of measures were extracted from each saccade. Saccade latency was defined as the amount of time between automatically defined end points of one saccade and the initiation of the next response. Saccade accuracy was examined using two measures to give an overall picture of spatial control: saccade amplitude: the extent of distance traveled from the start to the end point of the saccade; and distance error: the Euclidean distance of each saccade end point from its closest target. To show the control of saccade sequences across prior information level, data analysis was carried out and results are shown for averages of trial duration, saccade count, saccade latency, saccade amplitude and distance error across participant for each trial type. Each of these was subject to an analysis of variance and follow-up contrasts comparing each prior information with the previous one was carried out: 7 vs 5 targets; 5 vs 3 and 3 vs 1 target. Data in each figure are shown as the average across participants and error bars are within participants (Masson and Loftus 2003).

## Results

**Fig. 2:**
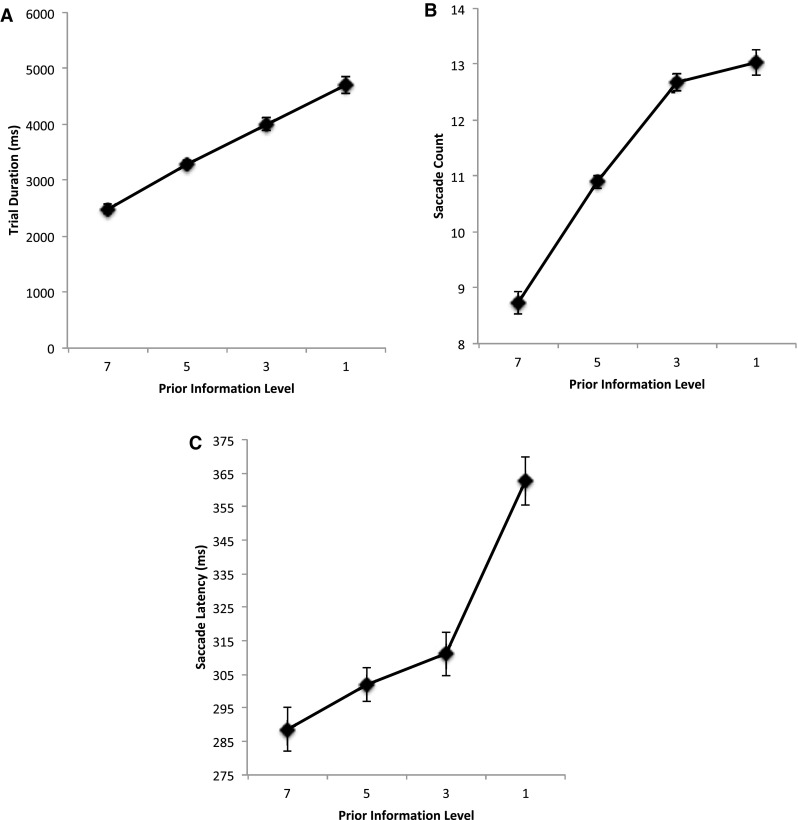
**a** Average trial duration, **b** average saccade count and **c** average saccade latencies (ms) as a function of restricting the availability of prior information about the location of the target positions. Information restriction increases from left to right, with locations known for all seven targets, the next five, three or one. Error bars are within participants’ error bars (Masson and Loftus 2003)

Figure 2 shows the average trial duration, saccade count and saccade latencies as a function of prior information about the target locations. From left to right on each graph in the figure, results show performance as the amount of prior information about the target locations is restricted from all seven targets displayed simultaneously, to only the next five targets displayed at a time, the next three targets are shown, finally to the next target only being revealed when a saccade was executed to the preceding target. This shows that, as prior information about the target locations was reduced trial duration increased, *F*(3, 51) = 52.5, MSE = 312,918, *p* < 0.001, *η*^2^ = 0.755 (with contrasts between sequential information levels, 7 vs 5, 5 vs 3 and 3 vs 1, showing significance at *p*’s < 0.011). This was a function of an increase in saccade count, *F*(3, 51) = 85.3, MSE = 0.818, *p* < 0.001, *η*^2^ = 0.834, and average saccade latency, *F*(3, 51) = 19.4, MSE = 977, *p* < 0.001, *η*^2^ = 0.532. Contrasts between each sequential level for saccade count show no significant difference between levels 3 and 1 (*p* = 0.30) but do show significance (*p*’s < 0.001) for 7 vs 5 and 5 vs 3. Contrasts for saccade latency also show a significant decrease in saccade latency as prior information increases (1 vs all other levels of prior information; 3 vs 7, *p*’s < 0.05; but not 5 vs 7). Having greater information about subsequent target locations improved task completion times through a combination of there being an overall reduction in the number of saccades being executed and reduced response latencies.^1^ It is worth noting that there was no difference in the first saccade latencies for each prior information level: prior information level 7, *M* = 318 (SD = 11); prior information level 5, *M* = 330 (SD = 27); prior information level 3, *M* = 317 (SD = 17); prior information level 1, *M* = 304 (SD = 17).

**Fig. 3:**
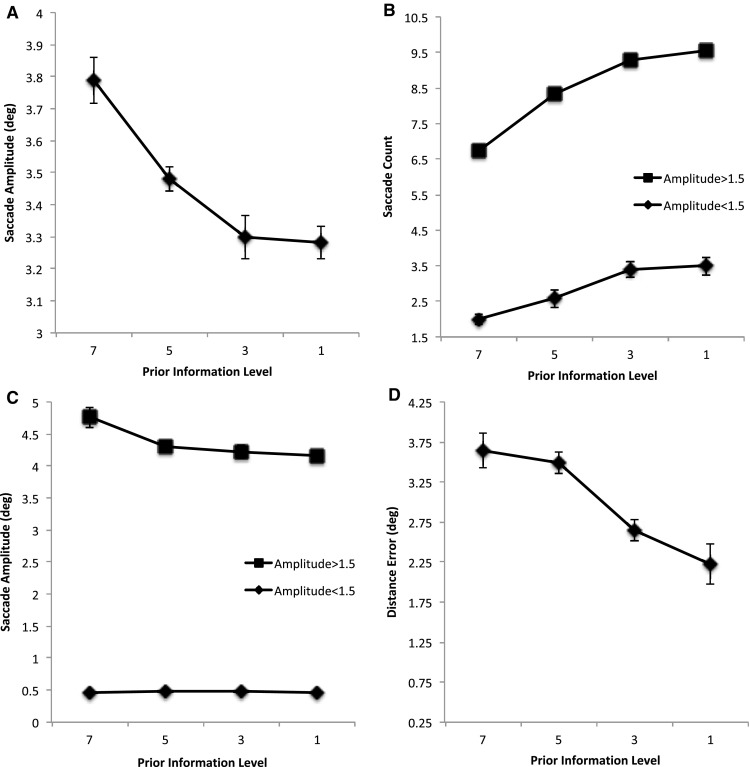
**a** Average saccade amplitude (degrees), **b** average saccade counts for short- and longer amplitude saccades and their **c** average saccade amplitudes (degrees). **d** Average saccade landing position error (Distance error) from nearest target location in degrees. All are shown as a function of restricting the availability of prior information about the location of the target positions. Error bars are within participants’ error bars (Masson and Loftus 2003)

Overall saccade amplitudes generally decreased as prior information about target locations decreases (Fig. 3a), *F*(3, 51) = 12.1, MSE = 0.083, *p* < 0.001, *η*^2^ = 0.416. Contrasts between each sequential level for saccade amplitude shows no significant different between levels 3 and 1 (*p* = 0.813) but start to show a trend toward significance between levels 5 and 3 (*p* = 0.079) and a significant difference between levels 7 and 5 (*p* < 0.001). Examination of the underlying distribution of amplitudes across participants shows a bimodal one with peaks at about 0.5° and 3° (not shown). These reflect target-driven saccades (the larger ones) and the corrective saccades (smaller or shorter ones). Separating amplitudes at the 1.5° trough between these peaks results in the graphs shown in Fig. 3b, c. Saccade counts can be seen to increase as prior information decreases for both the larger saccades and the smaller amplitude saccades (Fig. 3b; amplitude magnitude: *F*(3, 51) = 805, MSE = 1.41, *p* < 0.001, *η*^2^ = 0.979; prior information: *F*(3, 51) = 85.3, MSE = 0.409, *p* < 0.001, *η*^2^ = 0.834; interaction: *F*(3, 51) = 3.17, MSE = 0.267, *p* < 0.001, *η*^2^ = 0.411). While the interaction is significant, separate one-way ANOVAs for prior information depending on saccade amplitude continue to reveal significant effects for both large and small amplitude saccades: large amplitude saccades: *F*(3, 51) = 29.0, MSE = 0.390, *p* < 0.001, *η*^2^ = 0.814 and small amplitude saccades: *F*(3, 51) = 31.5, MSE = 0.286, *p* < 0.001, *η*^2^ = 0.650. Contrasts also show the same pattern across each amplitude magnitude with each level being significantly different from the next (all *p*’s < 0.01) for both the number of smaller and longer amplitude saccades. Average saccade amplitudes for large and smaller saccades (Fig. 3c) also show significant effects (amplitude magnitude: *F*(3, 51) = *F*(3, 51) = 2893.703, MSE = 0.188, *p* < 0.001, *η*^2^ = 0.994; prior information: *F*(3, 51) = 4.81, MSE = 0.128, *p* = 0.005, *η*^2^ = 0.220; interaction: *F*(3, 51) = 5.74, MSE = 0.128, *p* = 0.002, *η*^2^ = 0.252). Separate one-way ANOVAs depending on prior information show significant effects for the larger saccades, *F*(3, 51) = 5.34, MSE = 0.252, *p* = 0.003, *η*^2^ = 0.239, but no effect of prior information on the smaller ones, *F*(3, 51) = 1.046, MSE = 0.004, *p* = 0.380, *η*^2^ = 0.058. Contrasts showed no differences across information level for the smaller saccades (*p*’s > 0.148) but did show significant differences across information level for the longer amplitude saccades (7 vs 5 and 5 vs 3: *p*’s < 0.001; with levels 3 vs 1 also showing a trend, *p* = 0.058).

The average landing position error from the nearest target location is shown in Fig. 3d as a function of prior information. It can been seen that error reduces as the prior information is reduced reflecting the reduced influence of future target locations on saccade landing position control, *F*(3, 51) = 9.55, MSE = 0.874, *p* < 0.001, η2 = 0.360. Contrasts between each sequential level for saccade count shows no significant different between levels 7 and 5 (*p* = 0.268) but do show a significant difference (*p*’s < 0.001) for 5 vs 3 and a trend for 7 vs 5 (*p* = 0.058).

## Discussion

Previous work has shown that the processing of information about future saccade targets extended to at least the next three locations but whether it stretched beyond this was unclear. Using a task in which participants executed multiple saccades to a sequence of spots shows that parallel programming of saccade targets extends across the entire sequence. The time taken to complete the sequence reduced as more targets were available. This was due to both a decrease in number of saccades made and reduced latencies of these saccades. However, counter to this speed benefit came a poorer targeting of saccades. As more information about targets was made available, larger amplitude saccades (those that can be considered as being more target directed: those ≥ 1.5°) became longer. Interestingly, there were fewer shorter amplitude saccades, which maybe considered as reflecting corrective movements (< 1.5°), but these were equal in length to conditions in which less prior information about saccade targets was available. So, the less target location information is made available the more accurate the saccade is, likely reflecting a speed–accuracy trade-off. This shows the difficulty of executing saccades to isolated targets when they are in the presence of others. The influence of other target locations on saccade landing position control is more strongly felt the more information is available, with errors getting worse (control becoming more difficult) as more PI is available.

While we show a reduction in saccade latencies, as prior information about the target sequences increases, it is notable that this reduction is about 60–80 ms relative to executing saccades to targets presented individually (prior information of 1) but the latencies of the responses themselves are around 300 ms. This is strikingly longer than the very short latencies for secondary corrective saccades following initial error responses. Corrective saccades made to counter error responses in the anti-saccade task (Amador et al. 1998; Mokler and Fischer 1999; Weber et al. 1998) and the second saccades made in visual search tasks after distractor-directed saccades are corrected are shorter than those reported by about 200 ms than those reported here (Findlay et al. 2001; Godijn and Theeuwes 2002; Hooge and Erkelens 1996; McPeek et al. 2000; Theeuwes et al. 1998; Vivani and; Swensson 1982). It is argued that such very short inter-saccadic fixation periods found in the corrective saccade literature are possible if the second saccade has already been prepared. This is of course different to the demands that participants face in our task and could account for the different overall level of latencies reported. Indeed, our task is more similar to the two-step saccade task reported by Walker and McSorley (2006) in which participants make sequential saccades to two targets in turn. Walker and McSorley (2006) found that second saccade latencies were shorter relative to individual first saccade responses to the same targets (~ 80 ms) but the baseline second saccade latencies are of a similar magnitude to those reported here (around 300 ms), Similar to this, in our task, sequential target-directed saccades are required rather than error correcting secondary responses. It is likely that the underlying control structure that operates to control these is very different. In our task (and in Walker and McSorley 2006), multiple responses are being programmed concurrently. One possibility is that priority is given the next saccade and that subsequent saccades will be programmed to be less well-developed degrees. This is very different to corrective saccades made in response to an error which are assumed to be completely programmed prior to the onset of the initial error saccade.

There are at least two potential broad types of interpretations that could apply to these data which could be termed low level or high level, respectively. For the low-level interpretation, the results could be taken to show the impact on saccade control of each isolated target competing to become the next saccade target. As more targets become available, accuracy worsens as individual saccade control is influenced by the spatial locations of the others and speeded responses are made. On the other hand, a high-level interpretation is that participants adopt a strategy to follow the overall shape or Gestalt of the path of the spots rather than execute accurate isolated saccades to each target. Saccade accuracy worsens as they land, within the general shape and context of the path, between spots, and are less likely to be corrected. As a consequence, time taken to complete the trial decreases. On the other hand, an increase in visual information also results in the introduction of visual crowding. It is entirely possible that alongside Gestalt and overall path and shape processing that crowding leads to a change in the high-level strategy adopted by the participant. There may be a decrease in caution of the participant’s responses due to an increase in the uncertainty or difficulty of isolation of the individual target locations. This may result in a speeded and less accurately controlled sequence of saccades. Obviously these interpretations do not exclude the other. The results could reflect the operation of underlying processing mechanisms responsible for saccade location computation and the adoption of a higher level strategy due to general path of the shape or simply in response to the appearance of multiple new targets over a period of time.

Either of these types of explanations could take place within the context of the three-stage general framework for understanding eye movement control discussed in the Introduction. As outlined, this framework consists of three interconnected stages involved in the processing of goal directed saccades: a visual saliency stage in which bottom-up sensory encoding of stimuli takes place, the goal of which is to compute a saliency map (Itti and Koch 2000); an intermediate stage that combines saliency information with top-down goal demands and selection history to produce a common priority map of movement goals (Awh et al. 2012; Fecteau and Munoz 2006); and finally a motor stage in which motor representations are generated to produce eye movements. This saliency map computation could result in multiple points of saliency representing the saccade sequence which is folded into the priority map which could represent the top-down strategy to follow the shape or Gestalt of the shape (Awh et al. 2012; Fecteau and Munoz 2006; Godijn and Theeuwes 2002; McPeek et al. 2000) before finally feeding down to the generation of the motor map.

## Footnotes

1 In addition to the main experiment reported, two control experiments were carried out. The first examined whether the presence of numbers on each target rather than target spots alone impacted saccade control. The second examined whether the same patterns reported in the main experiment were also found in response to target sequences when targets were all displayed from the beginning of the trial. This directly tests whether the effects we report are due to the onset of successive new targets to the display (did they, for example, act as distracting stimuli in some way thereby affecting latency and accuracy). The first was carried out with five participants using the same methods as that reported in the main experiment but without numbers indicating the targets, i.e., the targets were unnumbered spots. The results were similar to that reported for the numbered targets suggesting that the numbers themselves were not responsible for parallel programming effects. As prior information (PI) about the target locations reduced trial duration increased [PI 7: 2425 (115); 5: 3292 (141); 3: 4440 (249); 1: 4361 (187) ms] with the effect saturating at prior information levels 3–1, *F*(3, 12) = 21.234, MSE = 216,339, *p* < 0.001, *η*^2^ = 0.841. Saccade count [PI 7: 9 (0.3); 5: 11 (0.2); 3: 12 (0.4); 1: 13 (0.2); *F*(3, 12) 25.316, MSE = 0.864, *p* < 0.001, *η*^2^ = 0.508] and saccade latency also increased [PI 7: 267 (7.5); 5: 309 (12); 3: 360 (9.6); 1: 345 (11) ms; *F*(3, 12) = 12.280, mse = 690.917, *p* < 0.001, *η*^2^ = 0.754]. Anecdotally, participants do prefer the numbered version of the experiment feeling more confident in target selection. In the second control experiment, we examined the potential role that visual transients and new target onsets may have played in the saccade latency reductions were report as prior information about the target sequences increases. 16 participants were required to saccade to target sequences in which no new targets were onset. Sequence lengths were of 7, 5 and 3 targets. If target onsets played role, we would expect to a change in the pattern of results we report in the main experiment. We found that saccade count and trial duration increased as the number of targets increased, as would expected. Importantly, the results also showed that average saccade latencies also decreased as sequence length increased. This suggests that the extent of parallel programming of saccades increased as target sequence length increased [PI 7: 302 (4.3); 5: 329 (3.8); 3: 384 (6.8); *F*(3, 12) = 43.7, MSE = 560, *p* < 0.001, *η*^2^ = 0.771].
